# Brain Sex in Transgender Women Is Shifted towards Gender Identity

**DOI:** 10.3390/jcm11061582

**Published:** 2022-03-13

**Authors:** Florian Kurth, Christian Gaser, Francisco J. Sánchez, Eileen Luders

**Affiliations:** 1 School of Psychology, University of Auckland, Auckland 1010, New Zealand; e.lueders@auckland.ac.nz; 2 Departments of Psychiatry and Neurology, Jena University Hospital, 07747 Jena, Germany; christian.gaser@uni-jena.de; 3 College of Education & Human Development, University of Missouri, Columbia, MO 65211, USA; sanchezf@missouri.edu; 4 Laboratory of Neuro Imaging, School of Medicine, University of Southern California, Los Angeles, CA 90033, USA; 5 Department of Women’s and Children’s Health, Uppsala University, 75185 Uppsala, Sweden

**Keywords:** brain, gender identity, machine learning, MRI, sex classifier, transgender

## Abstract

Transgender people report discomfort with their birth sex and a strong identification with the opposite sex. The current study was designed to shed further light on the question of whether the brains of transgender people resemble their birth sex or their gender identity. For this purpose, we analyzed a sample of 24 cisgender men, 24 cisgender women, and 24 transgender women before gender-affirming hormone therapy. We employed a recently developed multivariate classifier that yields a continuous probabilistic (rather than a binary) estimate for brains to be male or female. The brains of transgender women ranged between cisgender men and cisgender women (albeit still closer to cisgender men), and the differences to both cisgender men and to cisgender women were significant (*p* = 0.016 and *p* < 0.001, respectively). These findings add support to the notion that the underlying brain anatomy in transgender people is shifted away from their biological sex towards their gender identity.

## 1. Introduction

Transgender people report discomfort with their birth sex and a strong identification with the opposite sex. Transgender women are assigned male at birth but identify as female; transgender men are assigned female at birth but identify as male. Worldwide, the reported prevalence of transgender identities is rising [1], but our scientific understanding of how gender identity develops is still limited. Social explanations pointing to psychosocial and environmental influences [2,3] are complemented by biological explanations that include genetic predispositions and hormonal exposures [4,5,6,7,8,9]. Some (or perhaps all) of the aforementioned variables may have contributed to neuroanatomical variations in transgender brains, as repeatedly observed in both *post mortem* and in vivo studies published over the past three decades [10,11,12,13,14,15,16,17,18,19,20,21,22,23,24,25,26,27,28].

However, despite this wealth of research, a clear consensus is still missing in terms of which brain structures are altered in transgender individuals. Possible reasons include analyzing small and/or heterogeneous samples, applying different morphometric methods across studies, as well as focusing on single brain features. The latter is a concern in particular as even within cisgender studies there are large discrepancies in terms of reported sex differences, apart from the larger male and smaller female brain on average [29]. A possible solution is to study brain patterns rather than single features, as lately accomplished using modern machine learning algorithms in both cisgender samples [30,31,32,33,34] and transgender samples [35,36,37,38].

The overarching question addressed in those recent studies is whether the brains of transgender people are concordant with their birth sex or their gender identity, which is usually based on a so-called “classification accuracy” (i.e., how well can a brain be classified as male or as female). Interestingly, some studies [36,37] reported that the classification accuracy was reduced in transgender individuals, albeit not all studies observed this effect [35,36]. The reasons for divergences in study outcomes may be due to including individuals at different points in their gender-affirming process, using univariate classifiers and/or applying binary classifiers contrary to the notion that a mere binary classification may be insufficient to capture interactions between biological sex and gender identity [38].

The current study was designed to shed further light on the question of whether the brains of transgender people resemble their birth sex or their gender identity. For this purpose, we employed a recently developed multivariate classifier [34] that yields a continuous (rather than a binary) estimate for being male or female, in accordance with current biological models [39,40,41,42,43,44,45]. Our study sample consisted of 24 cisgender men, 24 cisgender women, and 24 transgender women before hormone therapy in order to rule out any modifying effects of circulating sex steroids [13,15,35,36,37,46,47,48,49,50,51,52]. We hypothesized that the estimated brain sex in transgender women is shifted away from their biological sex (male) towards their gender identity (female), but still significantly different from both.

## 2. Materials and Methods

### 2.1. Participants

Twenty-four transgender women (biological sex: male; perceived gender: female) were recruited through local community organizations and through professionals who offer services to the transgender community. To be included in this study, participants needed to self-identify as transgender women, report no history of hormone therapy, and declare the intention of undergoing estrogen replacement therapy. Moreover, participants were confirmed to be genetic males as defined by the presence of the SRY gene in their genome [53]. Six transgender women reported to be androphile (attracted to men) and 18 transgender women stated to be gynephile (attracted to women). The mean age of the transgender sample was 45.7 ± 13.8 years (range 23–72 years). The cisgender sample—selected from the International Consortium for Brain Mapping (ICBM) database (https://ida.loni.usc.edu/)—was close in age and handedness and consisted of 24 males (45.9 ± 13.7, 23–69 years) and 24 females (46.2 ± 14.0, 23–73 years). All participants provided informed consent, and ethics approval was granted by the Institutional Review Board of the University of California, Los Angeles (UCLA; protocol 041106703, 27 April 2007) and by the University of Auckland (UOA; protocol 022375, 30 November 2021).

### 2.2. Image Acquisition and Processing

All brain images were acquired on the same 1.5 Tesla MRI system (Siemens Sonata, Erlangen, Germany) using a T1-weighted sequence (MPRAGE) with the following parameters: TR = 1900 ms; TE = 4.38 ms; flip angle = 15°; 160 contiguous 1 mm sagittal slices; FOV = 256 mm × 256 mm; matrix size = 256 × 256, voxel size = 1.0 × 1.0 × 1.0 mm^3^. Brain images were processed using SPM8 (http://www.fil.ion.ucl.ac.uk/spm) and the VBM8 toolbox (http://dbm.neuro.uni-jena.de/vbm.html), as previously described [34,54,55,56]. In short, all brain images were tissue-classified into gray matter, white matter, and cerebrospinal fluid, and the resulting gray and white matter partitions were spatially normalized to MNI space using 12-parameter affine transformations. Finally, the normalized tissue segments were smoothed using an 8 mm FWHM kernel and resampled at 4 mm voxel size. These images constituted the input for the Brain Sex estimation.

### 2.3. Independent Training Sample

The Brain Sex classifier was trained on an independent set of MR images from an adult sample obtained from the IXI database (https://brain-development.org/ixi-dataset/). This training sample comprised brain scans from 547 adults (305 females/242 males) with an age range of 19 to 86 years (mean: 48.1 ± 16.6 years). All images from this training sample underwent the same preprocessing as described above for our current sample, resulting in smoothed normalized tissue segments resampled at 4 mm voxel size.

### 2.4. Data Reduction

Before training and running the classifier, a further data reduction step was performed as detailed elsewhere [34,54,55,56,57], using the Matlab Toolbox for Dimensionality Reduction (http://ict.ewi.tudelft.nl/~lvandermaaten/Home.html). In short, a principal component analysis (PCA) was run on the aforementioned independent training sample, and then the resulting transformations were applied to the study sample. Both PCA-transformed datasets, the independent training sample and the study sample, were then used as input for the Brain Sex classifier.

### 2.5. Brain Sex Estimation

Brain Sex was estimated using a Relevance Vector Regression (RVR) machine [58,59] within MATLAB (The MathWorks, Natick, MA, USA), as implemented in “The Spider” (https://people.kyb.tuebingen.mpg.de/spider/main.html). First, the classifier was trained on the aforementioned PCA-transformed independent training sample coding females as “0” and males as “1”. Then, the trained classifier was applied to the PCA-transformed study sample [54,55,57] generating the person-specific Brain Sex index—a number representing the degree of femaleness/maleness on a continuum (consistent with the training, a Brain Sex index of “0” signifies the average female brain and a Brain Sex index of “1” the average male brain).

### 2.6. Statistical Analysis

Before the main analysis, we assessed the classifier performance, both in the independent training sample (305 females/242 males) using a 10-fold cross-validation [34] and in our 48 cisgender brains (24 males/24 females). Specifically, the individual Brain Sex estimates were used to calculate the receiver operator characteristic (ROC) and its area under the curve (AUC). Furthermore, binarized estimates (female < 0.5; male ≥ 0.5) were used to calculate the classification accuracy (calculated as the number of true positives + the number of true negatives divided by the sample size) as quality metrics. For the main analysis, we applied an analysis of variance (ANOVA) comparing the 24 transgender women, the 24 cisgender men, and the 24 cisgender women. Significant effects were then followed up by one-tailed *post hoc* *t*-tests, in accordance with our hypothesis that brains of transgender women would be classified as less male-typical than brains of cisgender men but still more male-typical than brains of cisgender women. The effect sizes of these *post hoc* tests were calculated as Cohen’s d based on the difference in means and the pooled standard deviation. For all analyses, Bartlett’s and Lilliefors tests confirmed that the assumptions for parametric tests (i.e., equality of variance and normal distribution of the residuals, respectively) were met.

## 3. Results

**Figure 1:**
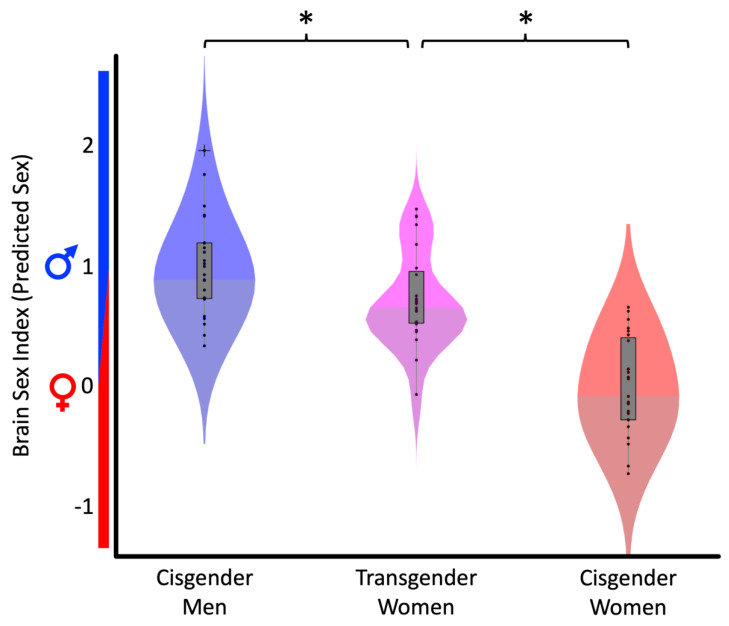
Significant Group Differences in estimated Brain Sex. The x-axis refers to the three groups. The y-axis displays the estimated Brain Sex (0 = average female; 1 = average male). Data are displayed as violin plots for cisgender men (blue), transgender women (pink), and cisgender women (red). The gray center of each violin contains the values between the 25th and 75th percentiles, the 24 black oval markers correspond to the 24 brains in each group, and the ‘+’ marks a brain that is outside the 1.5 interquartile range (vertical gray lines). The asterisks indicate significant group differences.

The classifier performed at 90.2% accuracy (AUC = 0.97) when assessed in the training sample and at 88.3% accuracy (AUC = 0.97) when assessed in our 48 cisgender brains. These measures indicate a suitable classification performance and a reliable distinction between the sexes based on brain anatomy. The estimated Brain Sex index was significantly different between the three groups (F(2,69) = 40.07, *p* < 0.001), with a mean of 1.00 ± 0.41 in cisgender men and of 0.00 ± 0.41 in cisgender women. The Brain Sex of transgender women was estimated as 0.75 ± 0.39, thus hovering between cisgender men and cisgender women, albeit closer to cisgender men (see also Figure 1). The follow-up post hoc tests revealed that transgender women were significantly more female than cisgender men (Cohen’s d = 0.64, t(46) = 2.20, *p* = 0.016), but significantly less female than cisgender women (Cohen’s d = 1.87, t(46) = 6.48, *p* < 0.001).

## 4. Discussion

The observed shift away from a male-typical brain anatomy towards a female-typical one in people who identify as transgender women suggests a possible underlying neuroanatomical correlate for a female gender identity. That is, all transgender women included in this study were confirmed to be genetic males who had not undergone any gender-affirming hormone therapy. Thus, these transgender women have been subject to the influence of androgens and grown up (at least up until a certain age) in an environment that presumably treated them as males. The combination of male genes, androgens, and (to some degree) male upbringing should ordinarily be expected to result in a male-typical brain [39,40,41,42,43,44,45], making a female-typical brain anatomy extremely unlikely. Yet, the brain anatomy in the current sample of transgender women is shifted towards their gender identity—an observation that is at least partly in agreement with previous reports, as discussed in the following.

Existing studies using multivariate classifiers aimed to assess whether the brains of transgender persons differ from their biological sex. For example, one study [37] investigated transgender men and transgender women before and after cross-sex hormone therapy using a binary classifier. The authors reported a significantly reduced classification accuracy in transgender persons compared to cisgender persons prior to hormone therapy, and the classification accuracy was even further reduced after therapy. This result might be explained by a shift in brain anatomy towards the gender identity (i.e., away from the biological sex), as also observed in the present study. Two other studies reported similar findings in transgender women but effects seemed to be driven by [36] or became significant only after [35] hormone therapy. Nevertheless, the sample sizes in those studies were extremely small (*n* = 8 and *n* = 11, respectively) and analyses were conducted using binary (rather than continuous) classifiers simply categorizing brains either as “male” or as “female”. Continuous classifiers (as applied in the current study) reflect a more nuanced classification by indicating where brains sit on the “male–female” spectrum but, to our knowledge, have not been used in transgender samples when analyzing structural magnetic resonance imaging (MRI) data. Nevertheless, even though findings are not immediately comparable, all existing structural MRI classifier studies—as well as a recent resting-state functional MRI classifier study [38]—seem to support the notion of a “shift” away from the biological sex towards the gender identity in transgender people. This shift has also been observed previously in some traditional region-of-interest studies focusing on single brain features and brain areas, such as the uncinate nucleus (INAH-3) [60], the insula and pars triangularis [14], the area around the central sulcus, posterior cingulate, and occipital regions [23] as well as the bed nucleus of the stria terminalis [22,28], just to name a few.

Future studies may further contribute to this field of research by replicating the current findings using continuous multivariate classifiers in independent samples. Ideally, those samples will be larger in size and include both transgender women and transgender men. Moreover, given that sexual orientation has been reported to affect brain anatomy [20,61,62,63,64,65,66], future studies might consider stratifying their transgender group(s), as well as their cisgender groups according to whether people are attracted to men, women, or both.

## Data Availability

Access to the cisgender data may be obtained via the International Consortium for Brain Mapping (ICBM) database (https://ida.loni.usc.edu/). Readers seeking access to the transgender data should contact the corresponding author.
